# Supplementary material for: Differential Magnitude of Resilience between Emotional Intelligence and Life Satisfaction in Mountain Sports Athletes
Source: Int J Environ Res Public Health. 2023 Aug 4;20(15):6525. doi: 10.3390/ijerph20156525 (PMC10419084; doi:10.3390/ijerph20156525)
Supplement: Supplementary file 1 [file ijerph-20-06525-s001.zip › ijerph-2500981-supplementary.pdf]

International Journal of
Environmental Research and Public Health

Supplementary Material

Differential magnitude of resilience between emotional intelligence and life satisfaction in mountain sports athletes

Guillermo Sanz-Junoy, Óscar Gavín-Chocano, José L. Ubago-Jiménez and David Molero

Supplementary Material

Factor loadings

From the data obtained with each of the instruments (Table Supplementary S1), a Confirmatory Factor Analysis (CFA) was performed to verify the validity and internal structure of each item. The factor loadings for the items of the EI scale (WLEIS-S), exhibited an adequate fit [62], $\chi 2/df$ = 2.856, with CFI = 0.962, SRMR = .0380, RMSEA = .0706. The reliability of this scale was Cronbach's α = .874 and McDonald's ω = .878.

Table Supplementary S1. Factor loadings WLEIS-S.

Latent factor	Indicator	α	ω	Estimate	*SE*	*Z*	*p*	β	*AVE*	*CR*
Appraisal of one's own emotions (SEA)	SEA 1	.862	.865	.791	.0123	64.1	< .001	.791	.645	.877
	SEA 2	.859	.862	.877	.0112	78.6	< .001	.904		
	SEA 3	.859	.862	.874	.0116	75.1	< .001	.878		
	SEA 4	.865	.870	.623	.0140	44.4	< .001	.603		
Appraisal of others' emotions (OEA)	OEA 5	.867	.873	.767	.0148	51.8	< .001	.713	.568	.839
	OEA 6	.867	.872	.975	.0160	61.1	< .001	.807		
	OEA 7	.872	.877	.716	.0159	45.2	< .001	.639		
	OEA 8	.863	.870	.898	.0140	64.2	< .001	.838		
Use of emotion (UOE)	UOE 9	.870	.874	.669	.0169	39.6	< .001	.546	.621	.863
	UOE 10	.865	.870	.893	.0161	55.5	< .001	.713		
	UOE 11	.859	.866	1.117	.0137	81.3	< .001	.921		
	UOE 12	.860	.866	1.109	.0139	80.0	< .001	.912		
Regulation of emotion (ROE)	ROE 13	.862	.865	1.135	.0116	98.1	< .001	.963	.596	.846
	ROE 14	.859	.862	.862	.0138	62.6	< .001	.760		
	ROE 15	.859	.862	.857	.0137	62.6	< .001	.518		
	ROE 16	.865	.870	1.128	.0115	98.1	< .001	.316		

Note: SE: Standardized error; *Z*: *Z*-value at estimation; *p*: *p*-value of Z-estimate; β: Standardized Estimate; AVE: Average Variance Extracted; CR: Critical Ratio.

The factor loadings for the items of the Resilience scale (RS-14), had adequate fit [62]; $\chi 2/df = 3.021$; with CFI = 0.901; SRMR = .0454; RMSEA = .0798. The reliability of this scale was Cronbach's α = .904 and McDonald's ω = .910 (See Table Supplementary S2).

Table Supplementary S2. Factor loadings RS-14.

Latent Factor	Indicator	*α*	*ω*	Estimate	*SE*	*Z*	*p*	*β*	*AVE*	*CR*
Personal competence (PC)	PC 1	.897	.904	.594	.0118	50.6	< .001	.667	.552	.877
	PC 2	.897	.900	.661	.0130	50.7	< .001	.668		
	PC 3	.898	.905	.784	.0160	49.1	< .001	.652		
	PC 4	.893	.904	.839	.0140	59.8	< .001	.754		
	PC 5	.900	.907	.755	.0168	44.8	< .001	.606		
	PC 6	.898	.899	.529	.0114	46.5	< .001	.624		
	PC 7	.901	.904	.601	.0145	41.3	< .001	.566		
	PC 8	.892	.904	.878	.0141	62.4	< .001	.777		
	PC 9	.898	.899	.584	.0124	47.2	< .001	.631		
	PC 10	.897	.909	.691	.0141	48.9	< .001	.650		
	PC 11	.894	.901	.656	.0107	61.1	< .001	.765		
Acceptance of self and life (ASL)	ASL 12	.905	.909	.699	.0185	37.9	< .001	.557	.504	.760
	ASL 13	.894	.904	.967	.0168	57.4	< .001	.810		
	ASL 14	.904	.900	.576	.0174	33.1	< .001	.496		

Note: SE: Standardized error; *Z*: *Z*-value at estimation; *p*: *p*-value of Z-estimate; *β*: Standardized Estimate; AVE: Average Variance Extracted; CR: Critical Ratio.

The factor loadings for the items of the Satisfaction with Life Scale (SWLS), had adequate fit [62]; $\chi 2/df = 3.243$; with CFI = 0.969; SRMR = .0587; RMSEA = .0696. The reliability of this scale was Cronbach's α = .883 and McDonald's ω = .903 (See Table Supplementary S3).

Table Supplementary S3. Factor loadings SWLS.

Latent Factor	Indicator	*α*	*ω*	Estimate	*SE*	*Z*	*p*	*β*	*AVE*	*CR*
Life satisfaction(LS)	LS 1	.832	.852	1.175	.0120	98.2	< .001	.986	.593	.852
	LS 2	.864	.895	.752	.0146	51.6	< .001	.658		
	LS 3	.832	.852	1.168	.0119	98.0	< .001	.939		
	LS 4	.857	.892	.768	.0148	51.9	< .001	.661		
	LS 5	.909	.915	.864	.0218	39.6	< .001	.530		

Note: SE: Standardized error; *Z*: *Z*-value at estimation; *p*: *p*-value of Z-estimate; *β*: Standardized Estimate; AVE: Average Variance Extracted; CR: Critical Ratio.
